# Changes in Sexual Risk-taking Behaviours Among Men After Participating in the PEST4MEN HIV Self-Testing Intervention in Two Fishing Communities in Central Uganda

**DOI:** 10.24248/eahrj.v9i1.824

**Published:** 2025-09-30

**Authors:** Joseph K.B. Matovu, Gloria Namazzi

**Affiliations:** a Department of Community and Public Health, Busitema University, Faculty of Health Sciences, Mbale, Uganda; b Department of Disease Control and Environmental Health, Makerere University School of Public Health, Kampala, Uganda.

## Abstract

**Background:**

The availability of free HIV self-test kits in the community may alter people's sexual behaviours in some way. However, little evidence exists to confirm or refute this assertion. We assessed changes in sexual risk-taking behaviours among men living in a fishing community before and after participating in an HIV self-testing (HIVST) intervention.

**Methods:**

This was a secondary analysis of data collected as part of a large peer-led HIVST intervention for men (PEST4MEN) in two fishing communities located in Kalangala (1) and Buvuma (1) Island districts. Following a baseline interview in July 2022, enrolled men (n=400) received oral fluid-based HIV self-test kits from their peer leaders and were followed up in September 2022 (n=361) to determine use. Data were collected on socio-demographic and behavioural characteristics using a structured questionnaire, configured in the KoboCollect tool, and loaded on mobile phones. We assessed changes in the proportion of men reporting multiple (2+) sexual partners, alcohol use before sex and condom use frequency before and after HIVST. We conducted descriptive analysis using STATA (version 14.0). Comparisons between proportions were made using Pearson's chi-square test.

**Results:**

Of 361 men, 239 had complete HIVST and sexual behaviour data at the baseline and follow-up visits. Of these, 34.3% (n=82) were aged between 25 and 34 years with a mean age of 30.8 years (Standard Deviation: ±9.0). Fifty-six percent (n=134) were engaged in fishing or fishing-related activities. The proportion of men reporting multiple sexual partners reduced significantly from 52.3% (n=125) to 42.3% (n=101), *P*=.0279. However, there was a non-significant increase in condom use at last sex (from 17.2%, n=41, to 18.4%, n=44; *P*=.7197) and alcohol use before sex (from 10.0%, n=24, to 11.7%, n=28; *P*=.5568). Consistent condom use reduced somewhat between the two study visits (from 10.0%, n=24, to 8.0%, n=19; *P*=.4241).

**Conclusion:**

The proportion of men reporting multiple sexual partnerships reduced significantly between the two study visits. However, this reduction was not observed in the other sexual risk behaviours. These findings suggest a need for integrating sexual risk-reduction messages into HIVST interventions in order to reduce sexual risk-taking behaviors among potential users of HIV self-test kits.

## BACKGROUND

HIV has unceasingly remained a global health burden, causing nearly 39 million deaths to date and 36 million morbidities.^1^ Despite the advancements in HIV treatment as prevention, many people still get infected with HIV with an incidence of 2 million infections per year.^1,2^ Sexual transmission remains the primary mode of HIV acquisition and transmission globally.^3^ Despite interventions to reduce sexual risk behaviours^4,5^, it seems likely that HIV transmission risk behaviour continues unabated even after people have tested for HIV and are aware of their HIV sero-status^6^, raising serious public health concerns as we move towards 2030, the magical year when the world intends to end HIV and AIDS as a global public health threat.^7^ While the emergence of HIV self-testing has helped to revolutionise the HIV testing landscape^8^, there are only a handful of studies that have examined if HIV testing has not resulted in sexual behavioural disinhibition.^9,10^ Our analysis aimed to fill this gap by examining changes in sexual risk-taking behaviours among men before and after using HIV self-test kits to generate data needed to design effective risk-reduction interventions among potential HIV self-test kit users.

HIV self-testing denotes the process of performing the HIV test on one's own, usually outside of formal health facility settings.^11^ A growing body of literature suggests that HIV self-testing is generally acceptable across settings and populations^12,13^ with the most commonly used kits being the oral fluid-based HIV self-test kits.^14^ HIV self-testing relies on the assumption that potential users can read and understand the information on the leaflets that are inserted into the HIV self-testing packages and that potential users can conduct the HIV self-testing exercise with minimal or no support at all.^15^ However, while this is correct, and many users have actually used HIV self-test kits on their own, the leaflets in the HIV self-testing packages do not contain information on what safer sexual behaviours HIV-negative testers should engage in (to remain HIV-free) or what HIV-positive testers should do to live positively with HIV. This absence of post-test counselling information may lead people to engage in risky behaviours that they would otherwise have avoided if such information were included in the leaflets.

Although evidence of harm, including suicide, after HIV self-testing is almost non-existent across studies and settings^16,17^, some studies show that HIVST may lead to undesirable sexual behaviours, including inconsistent condom use, an increase in the number of sexual partners or alcohol use before sex.^18^ For instance, a study conducted among female sex workers (FSW) in urban Kampala discovered that most FSW perceived HIVST as making condom-less sex safer, which could result in a decrease in condom use^19^. Similarly, a study conducted among university students in Uganda found that HIVST was favoured by students with multiple sexual partners and those who practised inconsistent condom use.^20^ In areas such as the fishing communities, which have high rates of transactional sex and sex without condoms, it is important to assess if HIVST could have led men to unintended changes in their sexual risk-taking behaviours.^21^ It is against this background that we assessed changes in sexual risk-taking behaviours among men before and after participating in a peer-led HIV self-testing intervention, hereafter referred to as PEST4MEN.

## METHODS

### Study Sites

This is a secondary analysis of data collected as part of a large, prospective, peer-led HIV self-testing intervention for MEN (PEST4MEN) in the Kalangala and Buvuma Island districts in central Uganda. The methods used in the PEST4MEN study have been described previously.^22,23^ In brief, the PEST4MEN intervention was implemented in two fishing communities located in the above-mentioned Island districts, within the Lake Victoria basin in central Uganda, between July and September 2022. Kalangala is one of the biggest Island districts in Uganda, with 84 islands and only 15 health facilities located on seven islands, serving a population of over 67,000 people. The highest-level health facility in Kalangala is Kalangala Health Centre IV, located on Buggala Island, the biggest island in the district. On the other hand, Buvuma, another Island district, consists of 52 islands with only 4 health centres located on four islands, serving a population of about 20,000 people. Given that the health facilities are located on just a handful of islands, access to HIV testing and other HIV services remains a challenge to people living in these Island districts. Our intervention aimed to bring HIV testing services closer to the population through promotion and distribution of HIV self-test kits within existing social/occupational groups of men. The study was conducted at Mwena fishing community in Kalangala district and Kasaali ‘B’ fishing community in Buvuma district. The selection of the two districts was based on their status as Island districts, the apparent lack of access to HIV testing services among a majority of the residents who live in far-off, remote fishing locations, away from the main health facilities^24,25^, and because the two districts have HIV prevalence levels that are higher than the average national HIV prevalence of 5.5% among adults aged 15 to 49 years.^26,27^

### Intervention Overview

The PEST4MEN intervention has been described elsewhere.^22,23^ In brief, the PEST4MEN intervention utilised eighteen male-only social/occupational groups to nominate twenty-two (22) peer leaders (kits distributors) through voting by raising hands during community meetings convened by members of the study team. A social network was defined as any loose grouping of men who lived or worked together and met daily or occasionally for social or economic purposes regardless of whether or not they engaged in fishing or fishing-related activities. One peer leader was selected from each social/occupational grouping following predefined selection criteria, including being able to keep secrets, being trustworthy (as judged by other members in the group) and being approachable. Peer leaders were required to be 18 years of age and above, residents of the fishing community, and literate in English and Luganda – given that they had key HIVST distribution tracking forms to complete. The selected peer leaders underwent a three-day training exercise on HIVST procedures, including checking test-kit expiry dates, opening the testing package and removing the test kit, using the test kit, opening the bottle with the testing solution and placing it in its stand, obtaining oral swabs, and timing the 20 minutes for the test. The training included demonstrations on how to use HIV self-test kits and how to read HIV self-test results by a study team member. The trained peer leaders were equipped with basic counselling/peer-to-peer counselling skills, and were taught about how to complete referral and study tracking forms and the importance of keeping HIV self-test results confidential, especially with regard to HIV-positive self-testers who may opt to disclose their HIV self-test results to them.

After the training, peer leaders were tasked to refer 20 members from their social/occupational groups, with whom they had regular weekly interactions and whom they knew at a personal level, for enrolment into the study. This was because the peer leaders had to physically deliver self-test kits, demonstrate HIV self-testing procedures, answer questions, and explain the next steps for those with HIV-positive results. The referred members were screened for eligibility, and a list of eligible participants was compiled and passed on to the peer-leader. Eligible social/occupational group members (see ‘study population’ below) completed a baseline questionnaire and were directed to their peer leaders to collect HIV self-test kits, as confirmed from the eligibility list shared with the peer leaders. They were asked to use the kits to self-test for HIV within one month and return the used kit to their peer-leader for safe custody or keep it and bring it to the study team at the next study visit for rereading by the study team.

### Study Population

This study was conducted among eligible male members selected by the trained peer leaders from their respective social/occupational groups, who were referred to the study team for eligibility screening. Eligible men had to be nominated by a trained peer leader from an existing social/occupational group, aged 15 years or older (15+ years), and self-report an HIV-negative or unknown HIV status. Men reporting an HIV-negative result at enrolment had to have last tested for HIV at least four months prior to study participation. Notably, the study only surveyed men referred by the peer leaders but not the peer leaders themselves.

### Data Extraction

Data were extracted from the main PEST4MEN study dataset. The PEST4MEN data were collected on men's socio-demographic and behavioural characteristics at baseline (July 2022) and at the first follow-up visit (September 2022), using a structured questionnaire, configured in *the KoboCollect* tool, and loaded on mobile phones. We extracted data on the study site, socio-demographic characteristics, sexual risk-taking and HIV self-testing behaviours. The data on the study site allowed us to stratify the analysis on sexual risk-taking behaviours by district of residence, while data on socio-demographic characteristics (e.g., age, marital status, occupation, and average monthly income) were extracted to provide a comprehensive understanding of the respondents’ characteristics. Data on the number of sexual partners, condom use frequency (i.e., always, sometimes/rarely, or never), condom use at last sex, and alcohol use before sex were extracted from the PEST4MEN dataset to compute changes in respondents’ sexual risk-taking behaviours before and after HIV self-testing. In addition, we extracted data regarding whether or not men used the HIV self-test kits that they received from their peer leaders. These data helped us to determine which men had self-tested for HIV and also to stratify the analysis on sexual risk-taking behaviours by men's HIV self-test results. Only those with complete data on these behaviours at both the baseline and follow-up visits were considered for further analysis. All variables were entered into STATA (version 14.0) for analysis.

### Measures

The primary outcome was men's self-reported change in sexual risk-taking behaviours after participating in the PEST4MEN intervention. Our measures focused on four primary behaviours: condom use frequency, condom use at last sex, number of sexual partners, and alcohol use before sex. Condom use frequency was coded as “always”, “sometimes/rarely” or “never” at both (baseline and follow-up) visits. Participants who reported that they used condoms in all sexual encounters (i.e., always) were considered to be consistent condom users. Therefore, only consistent condom use (“always”) was considered to be protective, while not using them at all (“never”) or using them “sometimes/rarely” was considered as engaging in sexual risk-taking behaviours. Condom use at last sex was defined as a dichotomous variable, with those who reported condom use at last sex categorised as “Yes” and those who did not as “No”. Individuals who reported condom use at last sex were considered to have engaged in protective sex. The number of sexual partners was categorised into “One sexual partner” or “2 or more sexual partners”. Men who reported 2+ sexual partners at each study visit were considered to have engaged in sexual risk-taking behaviours. Alcohol use before sex was measured as “always/most of the time”, “some of the time” or “never used alcohol” at both visits. Men who used alcohol before sex ‘always/most of the time’ and those who used alcohol before sex for ‘some of the time’ were considered to have engaged in sexual risk-taking sexual behaviours.

### Data Analysis

Descriptive analyses were conducted to determine the proportion of the respondents who engaged in sexual risk-taking behaviours before and after participating in the HIVST intervention. The analyses considered respondents with sexual partners at both baseline and follow-up surveys who reported that they self-tested for HIV. We determined if there was a change in sexual risk-taking behaviours between the baseline and follow-up visits and if that change tended towards protective or risky sexual behaviours. Due to small numbers, we were not able to conduct any regression analyses; however, comparisons between proportions were made using Pearson's chi-square test. Statistical analyses were computed in STATA, version 14.0.

### Ethical Approval

Ethical approval for the PEST4MEN study was sought from the Makerere University School of Public Health Research and Ethics Committee (Protocol #SPH-2021-158). The approved protocol was cleared by the Uganda National Council for Science & Technology as per national research guidelines (#HS2034ES). Written informed consent was obtained from all social network members. It is important to note that although we intended to enrol men aged 15+ years, we realized, at the time of analysis, that we did not have any men aged 15 to 17 years in the dataset.

## RESULTS

### Respondents’ Characteristics

This analysis focuses on 239 men with complete HIVST and sexual behavior data at the baseline and follow-up visits. Table 1 shows the characteristics of these men. In general, slightly more than two-thirds (67.4%; n=161) were aged below 35 years of age, and 67.8% (n=162) were currently married, while slightly more than half, 56.1% (n=134), were engaged in fishing or fishing-related activities. Slightly more than three-quarters (77.4%; n=185) of the men earned a monthly income ranging between UGX 101,000 and UGX 500,000 (~$27–$136; December 2024).

**TABLE 1:** Socio-demographics Characteristics of the Respondents

Variable	Frequency (n=239)	Percentage (%)
Age groups (years)		
18–24	79	33.0
25–34	82	34.3
35–44	58	24.3
45+	20	8.4
Mean age	30.8 (SD: ±9.0)	
Marital status		
Currently married	162	67.8
Ever married	25	10.4
Never married	52	21.8
Occupation		
Fishing/Fishing related activity	134	56.1
Other occupation (e.g. Farming, business)	105	43.9
Average monthly income (UGX)		
≤100,000	23	9.6
101,000–500,000	185	77.4
>500,000	31	13.0
Study site		
Kalangala (Mwena)	113	47.3
Buvuma (Kasaali-B)	126	52.7

### Overall Changes in Sexual Risk-Taking Behaviours Among Men Before and After Participating in the PEST4MEN Intervention

Table 2 shows men's engagement in sexual risk-taking behaviours before and after participating in the PEST4MEN intervention. Overall, the proportion of men reporting 2+ sexual partners dropped significantly from 52.3% (n=125) at baseline to 42.3% (n=101) at the first follow-up visit (*P*=.0279). There was a non-significant increase in the proportion of men reporting condom use at last sex from 17.2% (n=41) to 18.4% (n=44); *P*=.7197. The proportion of men reporting consistent condom use declined somewhat from 10.0% (n=24) at baseline to 8.0% (n=19) at follow-up. Meanwhile, there was a non-significant increase in the proportion of men reporting that they had never used a condom during any sexual encounter, from 54.4% (n=130) at baseline to 60.3% (n=144) at follow-up; *P*=.4241. The proportion of men who reported using alcohol before sex always/most of the time increased from 10.0% (n=24) at baseline to 11.7% (n=28) at the follow-up visit, but this increase was not significant, *P*=.5568.

**TABLE 2:** Overall Changes in Sexual Risk Behaviour, Before and After Intervention Participation

Variable	Before participating in the intervention n (%)	After participating in the intervention n (%)	*P-value*
Number of sexual partners			.0279
Only one	114 (47.7%)	138 (57.7%)	
2 and above	125 (52.3%)^*^	101 (42.3%)^*^	
Condom use frequency			.4241
Always	24 (10.0%)^*^	19 (8.0%)^*^	
Never	130 (54.4%)	144 (60.3%)	
Rarely/sometimes	85 (35.6%)	76 (31.8%)	
Condom use at last sex			.7197
No	198 (82.9%)	195 (81.5 %)	
Yes	41 (17.2%)	44 (18.4%)	
Alcohol use before sex			.5568
Always/Most of the time	24 (10.0%)^*^	28 (11.7%)^*^	
Never	149 (62.3%)	153 (62.0%)	
Some of the time	66 (27.6%)	58 (24.3%)	

The variables that are marked with an asterisk (*) are those that were compared to others using a Pearson's Chi square test.

### Sub-group analysis of sexual risk-taking behaviours before and after men participated in the PEST4MEN intervention

#### Sexual risk-taking behaviours stratified by district of residence

Table 3 shows results from a stratified analysis of sexual risk behaviours by district of residence. The proportion of men reporting 2+ sexual partners declined from 54.9% (n=62) to 46.9% (n=53) in Kalangala and from 50.0% (n=63) to 38.1% (n=48) in Buvuma, respectively. There were modest increases in self-reported condom use at last sex: in Kalangala, condom use at last sex increased from 22.1% (n=25) to 23.9% (n=27) in Kalangala and while in Buvuma, condom use at last sex increased from 12.7% (n=16) to 13.5% (n=17). However, consistent condom use declined from 18.9% (n=21) to 11.5% (n=13) in Kalangala but increased marginally from 2.4% (n=3) to 4.8% (n=6) in Buvuma. The proportion of men who reported using alcohol before sex always/most of the time increased from 11.5% (n=24) and 8.7% (n=11) at baseline to 12.4% (n=28) and 11.1% (n=14) at the follow-up visit in Kalangala and Buvuma, respectively.

**TABLE 3:** Changes in Sexual Risk-taking Behaviours by District of Residence, Before and After Intervention Participation

Variable	KALANGALA (N=113)			BUVUMA (N=126)		
	Before participating in the intervention n (%)	After participating in the intervention n (%)	*P-value*	Before participating in the intervention n (%)	After participating in the intervention n (%)	*P-value*
Number of sexual partners			.2311			.0570
Only one	51 (45.1%)	60 (53.1%)		63 (50.0%)	78 (61.9%)	
2 and above	62 (54.9%)^*^	53 (46.9%)^*^		63 (50.0%)^*^	48 (38.1%)^*^	
Condom use frequency			.1366			.3075
Always	21 (18.9%)^*^	13 (11.5%)^*^		3 (2.4%)^*^	6 (4.8%)^*^	
Never	49 (43.4%)	63 (55.8%)		81 (64.3%)	81 (64.3%)	
Rarely/sometimes	43 (38.1%)	37 (32.7%)		41 (33.3%)	39 (31.1%)	
Condom use at last sex			.7519			.8519
No	88 (77.9%)	86 (76.1%)		110 (87.3%)	109 (86.5%)	
Yes	25 (22.1%)^*^	27 (23.9%)^*^		16 (12.7%)^*^	17 (13.5%)^*^	
Alcohol use before sex			.8375			.5273
Always/Most of the time	13 (11.5%)^*^	14 (12.4%)^*^		11 (8.7%)^*^	14 (11.1%)^*^	
Never	57 (50.4 %)	66 (58.4%)		92 (73.0%)	87 (69.1%)	
Some of the time	43 (38.1%)	33 (29.2%)		23 (18.3%)	25 (19.8%)	

The variables that are marked with an asterisk (*) are those that were compared to others using a Pearson's Chi square test

#### Sexual risk-taking behaviours stratified by HIV self-test results

Table 4 shows the sexual behaviour of respondents based on HIV self-test results. Due to the small number of HIV-positive men, we focused mainly on the changes reported among HIV-negative men. Overall, the proportion of HIV-negative men reporting 2+ partners after self-testing significantly decreased from 53.4% (n=117) to 42.0% (n=92), *P*=.0168. Condom use at last sex increased from 16.0% (n=35) to 17.8% (n=39), while consistent condom use decreased from 9.6% (n=21) to 6.9% (n=15). There was a non-significant decrease in the proportion of men who reported using alcohol before sex for some of the time, from 26.0% (n=57) at baseline to 22.4% (n=49) at follow-up, *P*=.3721.

**TABLE 4:** Changes in Sexual Risk-taking Behaviours by HIV Self-test Results, Before and After Intervention Participation

Variable	Positive (N=20))			Negative (N=219)		
	Before participating in the intervention n (%)	After participating in the intervention n (%)	*P-value*	Before participating in the intervention n (%)	After participating in the intervention n (%)	*P-value*
Number of sexual partners			0.7491			0.0168
Only one	12 (60.0%)	11 (55.0%)		102 (46.6%)	127 (58.0%)	
2 and above	8 (40.0%)^*^	9 (45.0%)^*^		117 (53.4%)^*^	92 (42.0%)^*^	
Condom use with different sexual partners			0.6773			0.2966
Always	3 (15.0%)^*^	4 (20.0%)^*^		21 (9.6%)^*^	15 (6.9%)^*^	
Never	10 (50.0%)	11 (55.0%)		120 (54.8%)	133 (60.7%)	
Rarely/sometimes	7 (35.0%)	5 (25.0%)		78 (35.6%)	71 (32.4%)	
Condom use at last sex			0.7233			0.6100
No	14 (70.0%)	15 (75.0%)		184 (84.0%)	180 (82.2%)	
Yes	6 (30.0%)^*^	5 (25.0%)^*^		35 (16.0%)^*^	39 (17.8%)^*^	
Alcohol use before sex			1.0000			.3721
Always/Most of the time	1 (5.0%)	2 (10.0%)		23 (10.5%)	26 (11.9%)	
Never	10 (50.0%)	9 (45.0%)		139 (63.5%)	144 (65.8%)	
Some of the time	9 (45.0%)^*^	9 (45.0%)^*^		57 (26.0%)^*^	49 (22.4%)^*^	

The variables that are marked with an asterisk (*) are those that were compared to others using a Pearson's Chi square test.

#### Sexual risk-taking behaviours stratified by occupation

Table 5 shows changes in sexual risk-taking behaviours before and after men participated in the PEST4MEN intervention, stratified by occupation. Among men engaged in fishing/fishing-related activities, the proportion reporting 2+ partners decreased from 52.2% (n=70) to 41.8% (n=56), but consistent condom use and condom use at last sex declined between the two study visits. There was a marginal increase in the proportion of men reporting that they sometimes used alcohol before sex from 28.4% (n=38) to 29.9% (n=40). Among men reporting other occupations (other than fishing or fishing-related, e.g., those working in the palm oil plantations or other areas), the proportion reporting 2+ partners declined from 52.4% (n=55) to 42.9% (n=45), but consistent condom use declined between the two study visits. However, unlike men engaged in fishing or fishing-related activities, the proportion reporting condom use at last sex increased from 16.2% (n=17) to 21.0% (n=22). The proportion of men reporting that they always/most of the time used alcohol before sex increased from 7.6% (n=8) to 12.4% (n=13) over the two study visits.

**TABLE 5:** Changes in Sexual Risk-taking Behaviours by Occupation, Before and After Intervention Participation

Variable	Fishing/Fishing-related Activities (N=134)			Other Occupations (N=105)		
	Before participating in the intervention n (%)	After participating in the intervention n (%)	*P-value*	Before participating in the intervention n (%)	After participating in the intervention n (%)	*P-value*
Number of sexual partners			.0866			.1671
Only one	64 (47.8%)	78 (53.1%)		50 (47.6%)	60 (57.1%)	
2 and above	70 (52.2%)^*^	56 (41.8%)^*^		55 (52.4%)^*^	45 (42.9%)^*^	
Condom use frequency			.3734			.8181
Always	13 (9.7%)^*^	9 (6.7%)^*^		11 (10.5%)^*^	10 (9.5%)^*^	
Never	68 (50.8%)	79 (59.0%)		62 (59.1%)	65 (61.9%)	
Rarely/sometimes	53 (39.6%)	46 (34.3%)		32 (30.5%)	30 (28.6%)	
Condom use at last sex			.7459			.3749
No	110 (82.1%)	112 (83.6%)		88 (83.8%)	83 (79.1%)	
Yes	24 (17.9%)^*^	22 (16.4%)^*^		17 (16.2%)^*^	22 (21.0%)^*^	
Alcohol use before sex			.7880			.0952
Always/Most of the time	16 (11.9%)	15 (11.19%)		8 (7.6%)	13 (12.4%)	
Never	80 (59.7%)	79 (59.0%)		69 (65.7%)	74 (70.5%)	
Some of the time	38 (28.4%)^*^	40 (29.9%)^*^		28 (26.7%)^*^	18 (17.1%)^*^	

The variables that are marked with an asterisk (*) are those that were compared to others using a Pearson's Chi square test.

### Changes in condom use frequency and frequency of alcohol use before sex by self-reported number of sexual partners, before and after intervention participation

Table 6 shows the proportion of men who reported only one sexual partner and those who reported 2+ sexual partnerships at baseline by their condom use and alcohol use frequency before and after participating in the intervention. Among those reporting only one partner at baseline, consistent condom use increased from 5.3% (n=6) to 7.0% (n=8) after participating in the intervention, but condom use at last sex remained stable at 11.4%. The proportion of men who reported that they always used alcohol before sex increased from 4.4% (n=5) at baseline to 8.8% (n=10) after participating in the intervention. Among those who reported 2+ sexual partners at baseline, consistent condom use declined from 14.4% (n=18) to 8.8% (n=11), *p*=.1668, but the proportion reporting condom use at last sex increased slightly from 22.4% (n=28) to 24.8% (n=31), *p*=.6550. The proportion of men who reported always/most of the time using alcohol before sex decreased from 15.2% (n=19) to 14.4% (n=18) after men participated in the intervention.

**TABLE 6:** Changes in Condom Use Frequency and Alcohol Use Before Sex by Number of Self-reported Sexual Partners, Before and After Intervention Participation

Variable	Reported one sexual partner (N=114)			Reported 2+ sexual partners (N=125)		
	Before participating in the intervention n (%)	After participating in the intervention n (%)	*P-value*	Before participating in the intervention n (%)	After participating in the intervention n (%)	*P-value*
Condom use frequency			.5811			.1668
Always	6 (5.3%)^*^	8 (7.0%)^*^		18 (14.4%)^*^	11 (8.8%)^*^	
Never	92 (80.7%)	82 (71.9%)		38 (30.4%)	62 (49.6%)	
Rarely/sometimes	16 (14.0%)	24 (21.1%)		69 (55.2%)	52 (41.6%)	
Condom use at last sex			1.0000			.6550
No	101 (88.6%)	101 (88.6%)		97 (77.6%)	94 (75.2%)	
Yes	13 (11.4%)^*^	13 (11.4%)^*^		28 (22.4%)^*^	31 (24.8%)^*^	
Alcohol use before sex			.1817			.0985
Always/Most of the time	5 (4.4%)^*^	10 (8.8%)^*^		19 (15.2%)	18 (14.4%)	
Never	87 (76.3%)	78 (68.4%)		62 (49.6%)^*^	75 (60.0%)^*^	
Some of the time	22 (19.3%)	26 (22.8%)		44 (35.2%)	32 (25.6%)	

The variables that are marked with an asterisk (*) are those that were compared to others using a Pearson's Chi square test.

## DISCUSSION

This study assessed changes in sexual risk-taking behaviours among men before and after participating in the PEST4MEN HIV self-testing intervention in Kalangala and Buvuma Island districts in Uganda. Overall, study findings show that: a) there was a significant decline in the proportion of men reporting two or more sexual partners after participating in the intervention; b) a non-significant increase in condom use at last sex; c) a non-significant decline in consistent use of condoms; and d) a non-significant but slight increase in alcohol use before sex between the two study visits. These findings suggest that a reduction in the proportion of men reporting two or more partners was not followed with a similar positive change in other risk behaviours, suggesting ongoing HIV risk behaviours among the men studied.

### Interpretation of Findings and Comparison with Previous Studies

Our finding of a general decrease in the proportion of men reporting two or more sexual partners after participating in the intervention could be attributed to the increased health cautiousness among men who self-tested for HIV, especially those who received HIV-negative self-test results. However, these findings do not rhyme with those from similar studies; for example, in China, participation in HIV self-testing did not result in a reduction in the number of men who reported two or more sexual partners.^28^ A systematic review of HIV self-testing uptake and intervention strategies among men in Sub-Saharan Africa also did not show that participating in HIV self-testing resulted in any significant reductions in the number of sexual partners among individuals who self-tested.^18^ It is important to note that although men in our intervention reported a significant reduction in multiple sexual partnerships, we are not able to tell if the observed reduction was due to the intervention, given that the intervention did not aim to affect these behaviours in the first place. Further research is warranted to assess if participation in HIVST interventions can directly result in changes in sexual risk-taking behaviours. It is also important to note that when we stratified the reduction in multiple sexual partnerships by district of residence, we did not observe the same significant decline in the proportion of men reporting multiple sexual partnerships that we saw as part of the general analysis. This possibly suggests that there could be some form of confounding between multiple sexual partnerships and district of residence, although we were not able to tease this out due to small numbers.

We found that the proportion of HIV-negative men reporting two or more partners significantly decreased after participating in the intervention. Similar results have been reported in other studies. In a study conducted among female sex workers in Zambia, Oldenburg and others found that those who received HIV-negative results reported fewer partners than those who got reactive results.^29^ However, studies conducted in other settings, including China and the United States, found that receipt of HIV-negative self-test results was not significantly associated with a reduction in the number of sexual partners.^30,31^ While we may not be able to directly compare findings across studies due to differences in context and populations studied^30,31^, the lack of change in the number of sexual partners reported by men in other studies could be attributed to differences in the delivery of the interventions. Nevertheless, while our findings showed a significant reduction in the number of men reporting 2+ partners overall, this difference disappeared during the district-stratified analyses. It is also important to note that any other changes in sexual risk behaviours were insignificant. However, findings from other studies highlight that fishing environments constitute areas that continue to expose residents to ongoing risk behaviours ^32,33^ necessitating interventions to minimise these risks through targeted health promotion interventions. The differences in the observations could be attributed to the differences in the study population, where our study focused on only men, unlike other studies that included the general population in the fishing communities.

Although consistent condom use has been associated with reduced HIV risk in multiple settings ^34,35^, our findings show consistent condom use declined after men participated in the PEST4MEN intervention, including among HIV-negative men and those reporting 2+ partners. Despite the fact that our observations are based on small numbers, these findings are a cause of public health concern given that a reduction in consistent condom use among high-risk men, such as those reporting 2+ partners and men with HIV-negative status, can result in increased risks for HIV acquisition or transmission in the population.^36^ Our study findings are similar to those reported in one study in South Africa, which reported a decrease in consistent condom use with sexual partners.^37^ However, our results are inconsistent with the findings reported in another study in China, which reported that HIVST was associated with consistent condom use, more so among HIV-negative self-testers.^38^ This presents mixed results and calls for further research on this subject. Nevertheless, these observations suggest a need for integrating post-test counselling information into HIVST initiatives, including the need for consistent condom use, to increase the proportion of users who engage in protective behaviours after HIV self-testing.

In a subgroup analysis of men who reported two or more sexual partners at baseline, we found a decrease in consistent condom use coupled with a modest increase in condom use at last sex. It is important to note that the proportion of men who always/most of the time used alcohol before sex declined slightly by about 1%, while non-use of alcohol before sex increased by about 10% between the two study visits. Our findings are inconsistent with findings from other studies.^39,40^ In a study conducted among at-risk female sex workers in Kenya, Napierala and others ^39^, found a significant increase in condom use among their male sexual partners after HIVST, more so after a positive result^39^, and another study conducted in New York found reduced alcohol use among men who have sex with men after HIVST.^40^ The difference between our study and some of these studies could be due to the availability of post-test counselling services, which affirms the importance of integrating post-test counselling as part of HIVST processes. It could also be attributed to the differences in cultural, social, and environmental factors among the study populations in the different studies, as evidence shows that these also affect alcohol use.^41^ Taken together, these findings suggest that achieving changes in risk-taking behaviours among male fisherfolk may require more intense efforts and innovative/integrated approaches.

Generally, there was a minimal change in most of the sexual risk-taking behaviours among men after participating in the intervention. This could be partly attributed to the fact that the intervention was not designed to address sexual risk behaviours and partly to the lack of HIV status-specific post-test counselling information as part of HIVST initiatives. This observation calls for a need to integrate sexual risk-reduction messages into HIV self-testing initiatives in the future. Integrating sexual risk reduction messages into HIVST initiatives should help to inform HIV-negative self-testers what they should do to remain HIV-free and those with confirmed HIV-positive results how to live positively with HIV, including the benefits associated with immediate linkage to HIV care. At the moment, the information in the leaflets that are inserted in the HIV self-testing packages is restricted to HIVST user instructions, but no post-test counselling information is provided. This raises public health concerns given that post-test counselling has been shown to shape safe sexual behaviours among people living with HIV.^42,43^ Thus, in the future, it will be helpful to integrate HIV status-specific post-test counselling information into HIVST initiatives. However, since other studies have also reported notable increases in sexual risk behaviours after HIV self-testing^37,44^, further research is needed, possibly in a large prospective study, to assess if participating in an HIVST intervention may elevate sexual risk-taking behaviours among men or other populations.

### Study Limitations and Strengths

This study had some limitations and strengths. First and foremost, our analysis is based on small numbers that may not be sufficient to derive meaningful statistical computations. Thus, we were unable to conduct any deep-dive analyses to assess the reasons behind the observed changes in sexual risk behaviours. Secondly, sexual risk-taking behaviours were assessed as a secondary outcome; their assessment was not part of the primary objectives of the PEST4MEN intervention. Thus, our inability to see changes in most of the sexual risk-taking behaviours may be due in part to the fact that there was no deliberate effort to reinforce these behaviours as part of the PEST4MEN intervention and also in part to the fact that the study was not powered to assess changes in these behaviours. Thirdly, given that the findings are based on a single follow-up visit, we are unable to tell if the reported changes in sexual risk behaviours happened before or after the men participated in the PEST4MEN intervention. Thus, any attempts to attribute the observed changes in sexual risk behaviours to men's participation in the PEST4MEN intervention may not be feasible. Furthermore, given that we recruited socially-connected members of the peer-leaders, our findings may not apply beyond the settings studied. In addition, given that our analysis was based on self-reported changes in sexual behaviours, we cannot completely eliminate reporting bias. Nevertheless, the above-mentioned limitations notwithstanding, our study is among the few studies that have assessed sexual risk-taking behaviours among men following their participation in an HIV self-testing intervention. We believe that our findings can shed light on the need for integrating sexual risk-reduction messages into HIV self-testing interventions.

### Implications for Policy/Practice

Our study has important implications for policy and practice. First and foremost, our findings point to new research questions, including the need to examine the effect of post-test counselling information integrated into HIVST initiatives on sexual risk-taking behaviours among potential users of HIV self-test kits. Secondly, our findings point to the need for following up with men who have self-tested for HIV (and, by implication, other potential users) to support them to engage in positive behaviour change (e.g., consistent condom use or alcohol risk reduction), regardless of the type of results received. Thus, we believe that study findings will help to inform the design of future HIVST interventions targeting men in fishing community locations not only in Uganda but also in other settings in sub-Saharan Africa.

## CONCLUSION

We found a significant change in the proportion of men reporting 2+ sexual partners after participating in the PEST4MEN intervention, but this change was not replicated in the other sexual risk-taking behaviours. These findings suggest a need for integrating post-test counselling information (including on sexual partner risk reduction or consistent condom use) into the design and implementation of HIVST initiatives but also the need for continued follow-up support among HIV self-testers to reinforce safer sexual behaviours after HIV self-testing. Future studies, possibly large prospective studies, are warranted to confirm these findings.
